# A Focused and Efficient Genetic Screening Strategy in the Mouse: Identification of Mutations That Disrupt Cortical Development

**DOI:** 10.1371/journal.pbio.0020219

**Published:** 2004-08-17

**Authors:** Konstantinos Zarbalis, Scott R May, Yiguo Shen, Marc Ekker, John L. R Rubenstein, Andrew S Peterson

**Affiliations:** **1**Department of Neurology and the Ernest Gallo Clinic and Research Center, University of California at San FranciscoEmeryville, California, United States of America; **2**Loeb Medical Research Institute, University of OttawaOttawa, Ontario, Canada; **3**Nina Ireland Laboratory of Developmental Neurobiology, Department of PsychiatryLangley Porter Psychiatric Institute, University of California at San Francisco, San Francisco, CaliforniaUnited States of America

## Abstract

Although the mechanisms that regulate development of the cerebral cortex have begun to emerge, in large part through the analysis of mutant mice (Boncinelli et al. 2000; Molnar and Hannan 2000; Walsh and Goffinet 2000), many questions remain unanswered. To provide resources for further dissecting cortical development, we have carried out a focused screen for recessive mutations that disrupt cortical development. One aim of the screen was to identify mutants that disrupt the tangential migration of interneurons into the cortex. At the same time, we also screened for mutations that altered the growth or morphology of the cerebral cortex. We report here the identification of thirteen mutants with defects in aspects of cortical development ranging from the establishment of epithelial polarity to the invasion of thalamocortical axons. Among the collection are three novel alleles of genes for which mutant alleles had already been used to explore forebrain development, and four mutants with defects in interneuron migration. The mutants that we describe here will aid in deciphering the molecules and mechanisms that regulate cortical development. Our results also highlight the utility of focused screens in the mouse, in addition to the large-scale and broadly targeted screens that are being carried out at mutagenesis centers.

## Abstract 2

A novel screen uncovers thirteen mutants with defects ranging from the establishment of epithelial polarity in the cortex to the invasion of axons from the thalamus

## Introduction

The cerebral cortex is the seat of consciousness and the means by which we carry out abstract reasoning. Understanding how the cortex is assembled during embryonic development will give deeper insights into how this marvelous machine functions and provide the basis for therapy and repair. Although a diversity of approaches will be needed to answer all of our questions, an important starting point in studying events in development is often the careful analysis of mutant phenotypes. Much of what we know about cortical development has emerged through the study of mutations in mice and humans. For example, spontaneous mutations in mice such as *reeler* and *scrambler* have helped to tease apart the regulation of the radial migrations that create the cortical layers. Other important insights have come from the study of spontaneous mutations that cause radial migration defects and lead to lissencephaly and similar cortical defects in humans. Our understanding of radial migration and many other aspects of cortical development have also benefited enormously from the application of gene knockout approaches in mice. Despite this progress, many aspects of cortical development remain to be explored and would benefit enormously from additional mutant resources. The tangential migrations of cortical interneurons, for example, are regulated differently from the radial migrations of projection neurons, and only a few mutations have been described that disrupt interneuron migration.

Forward genetic approaches in the mouse, although technically feasible for many years, have become increasingly attractive with the availability of a dense genetic map and a nearly complete genomic sequence. These tools allow the process of gene identification, which was once very cumbersome, to be relatively straightforward. With the initial resurgence of interest in genetic screens, large-scale screens aimed at identifying mutations in broadly defined phenotypic categories were established at mutagenesis centers in Germany, the United Kingdom, the United States, and other countries (Hrabe de Angelis et al. 2000; Nolan et al. 2000). More recently, smaller, more focused screening efforts have had notable success (Vitaterna et al. 1994; Eggenschwiler et al. 2001; Kapfhamer et al. 2002; Garcia-Garcia and Anderson 2003; Hoebe et al. 2003), generally in situations where the effects of mutation on a specific cellular or biochemical process can be readily ascertained. Further development of forward genetics in the mouse as an approach with general utility will require the validation of focused screening strategies that allow for the identification of mutations disrupting specific processes in diverse situations, as has been done in other model organisms. In *Drosophila,* deletion strains and chromosomal inversions have been used to identify mutations within a specific region of the genome, and this approach has been elegantly adapted for use in the mouse (Juriloff et al. 1985; Kile et al. 2003). The development of screening strategies for the mouse that focus on the identification of mutations affecting a biological process rather than mapping to a certain genomic region will further expand the questions that can be addressed.

Here we describe a focused genetic screen in the mouse that takes advantage of a transgenic reporter line that labels the ganglionic eminences of the ventral forebrain and migrating cortical interneurons with β-galactosidase. The use of this reporter gene allows the isolation of mutations that alter growth and morphogenesis of the cortex very efficiently and, more specifically, allows the identification of mutations disrupting interneuron migration from the ganglionic eminences into the cortex. In this screen, we isolated four mutations that affect tangential migration. We also isolated nine novel mutations affecting other aspects of cortical development; these mutations include three that represent novel alleles of genes that have been shown to have a role in cortical development. These results illustrate the power of a forward genetic approach, borrowed from other model organisms, that can be applied to various questions of mammalian biology.

## Results

### The *Dlx-LacZ* Transgene Labels the Ganglionic Eminences and Migrating Interneurons

**Figure 1:**
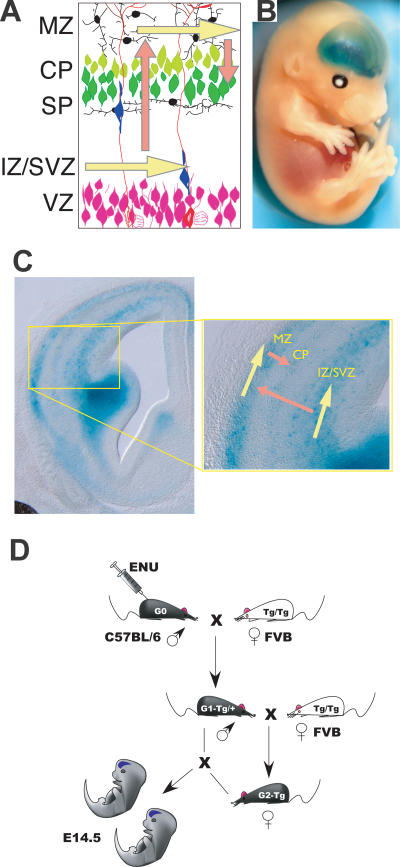
The Strategy for Isolation of Recessive Mutations Disrupting Cortical Development (A) Migrating interneuron precursors appear to migrate tangentially (yellow arrows) through both the MZ and the IZ/SVZ. Precursors also migrate radially (pink arrows) to reach the developing cortical plate (CP). (B) Migrating interneuron precursors expressing β-galactosidase can be seen in a whole-mount preparation of an E14.5 Dlx-LacZ mouse as diffuse cortical staining. (C) In coronal section, the densely labeled SVZ of the LGE can be seen, as can the streams of migrating interneuron precursors in the cortex. (D) To identify recessive mutations, male C57BL6/J mice were treated with ENU and then crossed to FVBN/J females that were homozygous for the *Dlx-LacZ* transgene. Male offspring of this cross were backcrossed to produce female offspring that were then backcrossed to their fathers. Embryonic litters from these backcrosses were stained and examined for defects at E13.5 or E14.5.

The expression of Dlx family homeodomain transcription factors is necessary for differentiation of neurons in the striatum and for migration of many, if not all, of the interneuron precursors that arise in the ganglionic eminences of the embryonic telencephalon (Marin and Rubenstein 2001, 2003). A transgenic line in which the expression of β-galactosidase is driven by transcriptional control sequences from the *Dlx5/6* intergenic region faithfully recapitulates most aspects of Dlx5 expression in the central nervous system, including strong expression in the subventricular zone (SVZ; a secondary progenitor zone) of the ganglionic eminences, in immature interneurons as they migrate tangentially through the intermediate zone (IZ) or marginal zone (MZ) of the cortex (Figure 1A–1C), and in mature cortical GABAergic interneurons (Stuhmer et al. 2002). The dispersed interneuron precursors label the cerebral cortex, and the underlying high-level expression in the ganglionic eminences provides a useful adjunct to the morphological landmarks, allowing cortical defects to be identified in whole-mount stained embryos. We took advantage of the Dlx-LacZ line as a background upon which to screen for mutations. Mutations were induced using ethyl-nitroso-urea (ENU), a mutagen that induces primarily single-base substitutions with very little bias (Russell et al. 1979; Vrieling et al. 1988; Nivard et al. 1992), and animals were bred in order to detect recessive mutations that disrupted the distribution of Dlx-LacZ–labeled cells in the developing cortex at embryonic day 14.5 (E14.5) (Figure 1D). In total, 705 litters with an average of seven embryos each, and representing 305 lines of mice, were stained using β-galactosidase histochemistry, and then examined in whole mount; higher-resolution analysis on 100-μm coronal vibratome sections of brains was also performed on a third (225) of the litters.

### Dlx-LacZ Allows Efficient Identification of Cortical Mutants

**Figure 2:**
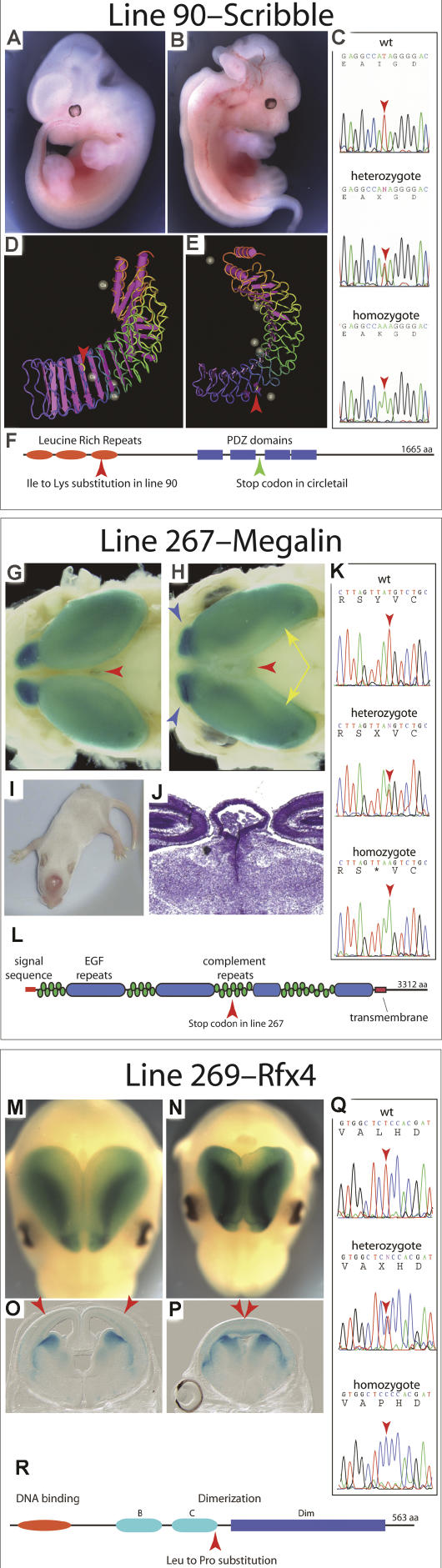
Novel Alleles of *scribble, megalin,* and *Rfx4* (A and B) Comparison of E12.5 WT (A) and mutant (B) embryos showing the severe craniorachischisis of line 90 embryos. (C) Sequencing chromatograms show the thymine in the WT sequence (top) that is an adenine in line 90 (bottom). This thymine-to-adenine substitution changes an isoleucine codon to a lysine codon within a conserved LRR domain of SCRIBBLE. (D and E) Ribbon diagrams of an LRR fold, illustrating the predicted position (red arrowheads) of the line 90 amino acid substitution. The substitution is in a linker region between two stretches of β-sheet. An LRR fold has a β-sheet on the inside, and linker regions on the outside, of a broad half-circular curve. (F) The predicted domain structure of SCRIBBLE indicating where the line 90 and the *Circletail* alleles alter the protein relative to the three LRR and four PDZ domains. (G and H) Dorsal views of the cortex of E17.5 WT (G) and line 267 mutant (H) embryos stained to reveal the expression of the *Dlx-LacZ* transgene. The red arrowheads point to the choroid plexus of the third ventricle, which is stained because of its endogenous β-galactosidase expression, and which is greatly enlarged in the mutant. The cortex of the mutant is longer along the rostrocaudal axis and is altered in shape in the caudal portion (yellow arrows). The olfactory bulbs (blue arrowheads) are also altered in shape. (I) The choroid plexus persists in its hypertrophic state after birth and can be seen as a pinkish lump on the head of this postnatal day 14 (P14) pup. (J) A coronal section through the dorsal portion of the diencephalon of a P0 pup. (K) Sequencing chromatograms show the thymine in the WT sequence (top) that is an adenine in line 267 (bottom). (L) The predicted domain structure of MEGALIN indicating where the new stop codon is introduced by the line 267 mutation. (M and N) Dorsal views of the cerebral cortex of E14.5 WT (M) and line 269 mutant (N) embryos stained to reveal the expression of the *Dlx-LacZ* transgene. (O and P) Coronal sections of E14.5 WT (O) and line 269 mutant (P) forebrains. Red arrowheads indicate the apparent span over which dorsal midline structures are lost in the mutant. (Q) Sequence chromatograms showing the thymine in the WT sequence (top) that is a cytosine in line 269 (bottom). (R) The predicted domain structure of RFX4 indicating where the line 269 substitution alters the protein relative to the DNA binding and the extended (B, C, and Dim) dimerization domains.

During the course of screening we identified eight mutants with defective growth or patterning of the cerebral cortex, four mutants with defects in the migration of interneuron precursors into the cortex, and one mutant in which thalamocortical axons fail to invade the cortex (Table 1). Lines of mice carrying each mutation were established, in which the phenotype was propagated as a recessive trait with Mendelian inheritance. Preliminary mapping, together with the overt phenotype, pointed us toward a likely locus for three of the mutants (Figure 2). The other ten mutations appear to identify loci whose role in forebrain development has not previously been described.

**Table 1:**
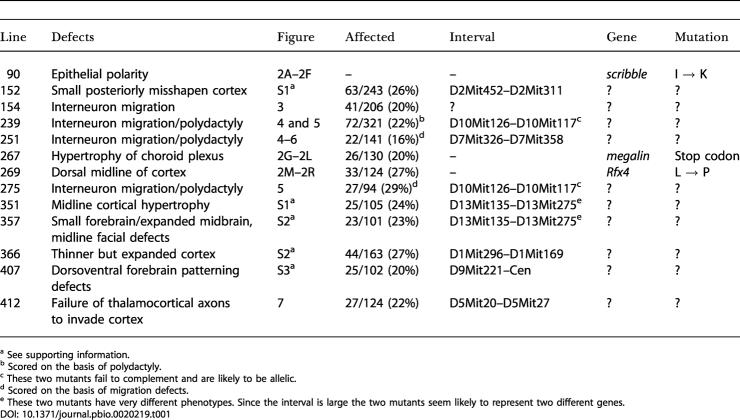
Summary of Cortical Mutants

^a^ See supporting information

^b^ Scored on the basis of polydactyly

^c^ These two mutants fail to complement and are likely to be allelic

^d^ Scored on the basis of migration defects

^e^ These two mutants have very different phenotypes. Since the interval is large the two mutants seem likely to represent two different genes

### A Novel *scribble* Allele

The three mutations in previously characterized loci produce alleles that differ from the existing ones in informative ways (Figure 2). Mice homozygous for the line 90 mutation have an open neural tube in the spinal cord and hindbrain region, or craniorachischisis, and a disorganized and hyperplastic neuroepithelium in the cortex and other parts of the central nervous system (Figure 2A and 2B). An essentially identical phenotype is seen in homozygous *Loop-tail and Circletail* mutants (Kibar et al. 2001; Murdoch et al. 2001a, 2001b, 2003), both of which also have dominant tail defects, a phenotype that is not seen in line 90. Both *Loop-tail* and *Circletail* mice have mutations in genes that regulate planar cell polarity. *Loop-tail* mice carry a mutation in the *strabismus-1* gene *(Str-1,* also known as *Ltap/Lpp1),* and *Circletail* mice have a mutation in the *scribble* gene *(Scrb1).* Mapping of the line 90 mutation places it on Chromosome 15 in the region of *Scrb1. Scrb1* encodes a protein of 1,665 amino acids with three leucine-rich repeat (LRR) domains near the amino terminus and four centrally located PDZ domains (Figure 2F). Sequencing of RT-PCR products from line 90 mice identified a missense mutation in *Scrb1* that causes an isoleucine-to-lysine substitution in the third LRR domain (Figure 2C–2F).

In *Drosophila,* a critical role for a *Scrb1* homolog, *scribbled,* in establishment of apical basal polarity in epithelia has been described (Bilder and Perrimon 2000; Bilder et al. 2000, 2003). Analysis of mice carrying the *Circletail* allele of *Scrb1* show a loss of planar cell polarity in the hair cells of the inner ear, indicating that *Scrb1* is required for the establishment of planar cell polarity rather than apical-basal polarity in the mouse (Montcouquiol et al. 2003). Interestingly, a strong genetic interaction has been observed between the *Circletail* allele of *Scrb1* and *Loop-tail* mutants (Murdoch et al. 2001b). The interaction is strong enough that compound heterozygotes for the two mutations show a severe phenotype that is indistinguishable from the individual homozygous phenotypes. The molecular nature of the *Circletail* allele of *Scrb1,* with a frameshift mutation that causes a premature stop codon after the first two PDZ domains (Murdoch et al. 2003), is substantially different from that of line 90. To see whether interaction between the two loci was a unique attribute of the *Circletail* allele, we crossed line 90 with *Loop-tail* mice. As with the *Circletail* allele, a strong genetic interaction was seen between the line 90 allele of *Scrb1* and the *Ltap/Lpp1* mutation, such that embryos indistinguishable from homozygotes of the individual mutations were recovered (unpublished data). This indicates quite clearly that the integrity of the LRR domains is critical for the tight coordination between *Scrb1* and *Ltap/Lpp1* in establishment of planar cell polarity.

### A Novel *megalin* Allele

We also identified a mutation that caused an enlarged cortex, hypertrophy of the choroid plexus of the third ventricle, and abnormalities in the dorsal diencephalon (Figure 2G–2J). Mapping of this mutation places it on Chromosome 2 in a region containing the *megalin* gene (also known as *Lrp2*). Prolapse of the third ventricle choroid plexus was described in a knockout allele of *megalin* (Willnow et al. 1996), suggesting that this might be the responsible locus in this case. On this basis we sequenced RT-PCR products from the *megalin* gene and identified a base substitution producing a premature stop codon rather than the tyrosine codon at residue 2721 (Figure 2K and 2L). The ENU-induced allele is predicted to express a truncated MEGALIN protein consisting of the amino-terminal portion of the extracellular domain, whereas the knockout is a null. Unlike the case of *Scrb1,* where the phenotypes produced by the two alleles are essentially identical, the two *megalin* alleles show phenotypic differences. Although both alleles produce a hypertrophic choroid plexus, this defect is more pronounced with the ENU-induced allele. We have found that the choroid plexus defects are also associated with an expansion and inhibition of differentiation in the dorsal neuroepithelium of the diencephalon that would ordinarily form the subcommissural organ and of the pineal gland just caudal to the defective choroid plexus (see Figure 2I–2J; A. Ashique, unpublished data). It is not possible to say from the published characterization of the knockout allele whether a similar condition occurs there, but it is clear that the knockout allele causes holoprosencephaly (Willnow et al. 1996), a phenotype that we have not seen in line 267. Indeed, the ENU-induced allele causes an enlarged cortex (Figure 2G and 2H) without any obvious deficiency of midline structures such as would be expected in even mild holoprosencephaly.

### A Novel *Rfx4* Allele

A morphologically identifiable dorsal midline is absent from the cerebral cortex of line 269 homozygotes (Figure 2M–2P). This is an unusual defect that is similar to that seen in a transgene insertion mutation that disrupts the *Rfx4* gene (Blackshear et al. 2003). The line 269 mutation was mapped to Chromosome 10 in the region containing *Rfx4*. Sequencing of *Rfx4* revealed a base substitution that changes an evolutionarily conserved leucine residue to proline (Figure 2Q and 2R). RFX4 is a member of the Rfx subfamily of winged-helix transcription factors (Emery et al. 1996) and can form dimers with two of the other family members, RFX2 and RFX3 (Morotomi-Yano et al. 2002). The amino acid substitution affects the conserved C domain of RFX4's large dimerization domain (Katan-Khaykovich et al. 1999). The transgene insertion mutation, on the other hand, selectively eliminates the expression of a neural-specific transcript of the gene, and so behaves as a tissue-specific null (Blackshear et al. 2003). Dimerization is not necessary for DNA binding activity of Rfx-class transcription factors (Katan et al. 1997; Katan-Khaykovich and Shaul 1998). Instead, dimerization appears to determine whether the bound transcription factor mediates transcriptional activation or repression. The fact that the line 269 allele produces a phenotype that is apparently identical to that of a null allele provides clear evidence that RFX4-containing dimers regulate important transcriptional regulatory events during formation of the cortical midline.

### Dlx-LacZ Allows Efficient Identification of Tangential Migration Mutants

Four mutants were identified in which the morphology of the cortex was normal at E14.5, but in which the distribution of LacZ-expressing cells in the cortex was altered. Three mutants, 154, 239, and 275, were identified on the basis of defects that are apparent in whole-mount preparations. Defects in the fourth mutant, 251, were only apparent upon examining sections. All four mutations are, for unknown reasons, perinatal lethal when homozygous, and this has prevented us from analyzing adult phenotypes. In addition, the line 154, 239, and 275 mutations appear to cause an increase in spontaneous seizures and mortality in young adult heterozygotes. Because the seizures are sporadic, their basis has not yet been studied.

**Figure 3:**
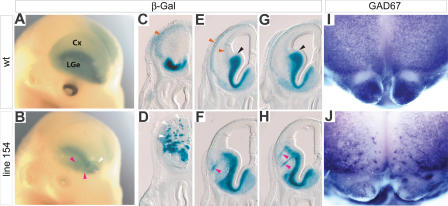
Severe Disruption of Interneuron Migration in Line 154 Mutants (A) Disseminated immature interneurons are seen as diffuse cortical (Cx) staining in WT embryos. Subcortical expression in the SVZ of the LGE can be seen as a darkly stained, inverted crescent. (B) Embryos homozygous for the line 154 mutation have little or no cortical staining, and the subcortical staining has aberrant streaks (pink arrowheads) and spots (white arrowhead), particularly in the frontal cortex. (C, E, and G) In this rostral-to-caudal series of coronal sections from WT embryos, the normal MZ and IZ/SVZ migratory streams are diffusely labeled (red arrowheads), and a sharp cortical-subcortical boundary (black arrowheads) is marked by the abrupt transition between the densely stained SVZ of the LGE and the diffuse staining of the migrating interneuron precursors. (D, F, and H) A rostral-to-caudal series of coronal sections from a line 154 mutant embryo shows the rostral spots (white arrowheads) visible in whole mount to be aggregates of cells in the cortex, and the streaky subcortical staining to be radially directed linear aggregates (pink arrowheads). The SVZ of the LGE is also noticeably thinner, and there is not a well-defined cortical-subcortical transition in the staining pattern. (I and J) Rostral views of *Gad67* whole-mount in situ hybridization show the pattern of migration abnormalities in the cortex and significant defects in population of the olfactory bulb by GABAergic neurons.

Line 154 has the most severe defects. Affected embryos often have fewer labeled cells in the cortex except in the most rostral regions, where they form abnormal aggregates (Figure 3A and 3B). Abnormalities in the LacZ expression pattern that are associated with the failure of cells to invade the developing cortex can also be found in the subcortical telencephalon of line 154 embryos (Figure 3C–3H). Here, prominent, radially oriented columns of cells form near or at boundaries between subcortical subdivisions: the dorsal LGE, the ventral LGE, and the medial ganglionic eminence. To confirm that the abnormal pattern of LacZ expression was due to a perturbation in the distribution of interneurons in the cortex and not the result of ectopic expression of the transgene, we examined the expression of an interneuron marker, *glutamic acid decarboxylase 1 (Gad67).* Interneurons are GABAergic and so express GAD67, an enzyme involved in GABA synthesis. Glutamatergic projection neurons do not express GAD67. Whole-mount in situ analysis of GAD67 at E15.5 showed that the distribution of interneurons was abnormal, as predicted by the aberrant expression of the transgene (Figure 3I and 3J).

### Three Migration Mutants Have Defects in a Common Process

**Figure 4:**
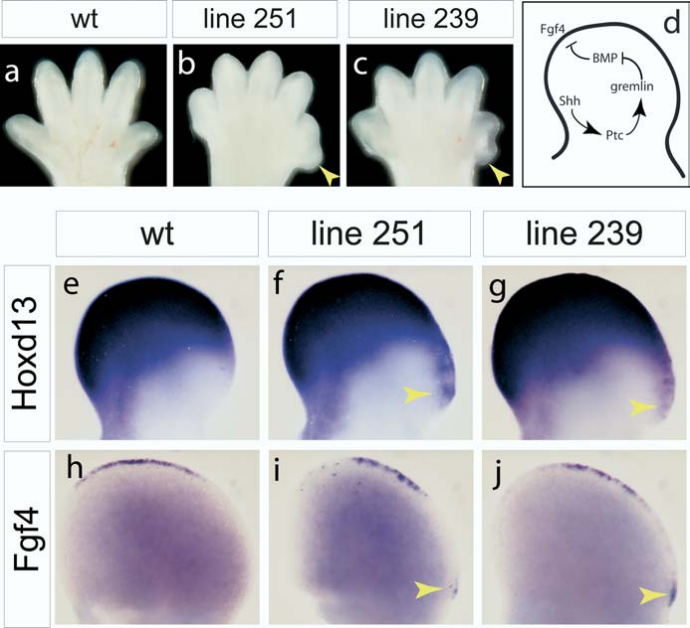
Limb Patterning Defects in Tangential Migration Mutants Anterior is to the right for all limbs. (A–C) Left hindlimbs are shown from E14.5 WT (A), line 251 (B), and line 239 (C). Yellow arrowheads point to extra digits on the anterior (thumb) side of the limb in mutants. Line 275 mutants also have anterior polydactyly. The mutants have a slight developmental delay that causes some differences in appearance of the limb buds at E14.5, when the limbs are growing rapidly. (D) This diagram illustrates components of the *Shh/Fgf* feedback loop that maintains *Fgf4* expression in the AER. (E–J) In situ hybridization on E11.5 limb buds. Unlike in WT (E), expression of the posterior patterning gene *Hoxd13* in left forelimb buds extends ectopically into an anterior domain (yellow arrowheads) in 251 (F) and 239 (G) mutants. *Fgf4* expression in left hindlimb buds, restricted to the posterior AER in WT (H), has an ectopic expression domain at the far anterior edge of the AER (yellow arrowheads) in 251 (I) and 239 (J) mutants.

Lines 239, 251, and 275 share features that suggest that the loci involved may have roles in a common regulatory process. All three mutations produce defects that are most apparent in the rostral cortex at E14.5, and all three cause anterior polydactyly (Figure 4A–4C; unpublished data). The limb defects might be only superficially similar or they could have a common developmental basis. If the latter explanation were true, it would strongly suggest that the neuronal migration phenotypes of the three mutants are similar because the same regulatory mechanism is defective in all of them. Initial mapping uncovered linkage to Chromosome 7 for the line 251 mutation and to Chromosome 10 for the other two, indicating that at least two distinct loci are involved (Table 1). To determine the number of loci involved, complementation tests were carried out using crosses between all three lines. The line 251 mutation complemented the other two as expected, but crosses between line 239 and line 275 produced mutant embryos with both limb and forebrain defects that were indistinguishable from either parental line. Further study will be required to determine whether the two lines carry the same or two independent mutations.

To identify the developmental basis of the limb defects, we examined the expression of genes involved in limb patterning. A regulatory network involving *Shh* and *Fgf4* regulates the expression of *Hoxd13* and controls patterning of the limb (Figure 4D). We first investigated the expression of *Hoxd13* as a molecular marker of anterior-posterior (A-P) pattern in the limb at early stages. *Hoxd13* is ectopically expressed in the anterior portion of line 239 and line 251 mutant limbs (Figure 4E–4G), indicating that the anterior polydactyly is caused by a defect in A-P patterning. We next examined the expression of the elements of the network that regulate *Hoxd13* expression (Figure 4D) to see whether their expression was perturbed. The expression of *Shh*, *Ptc1, gremlin* (also known as *Cktsf1b1*), and *Bmp4* are not distinguishable from wild-type (WT) (unpublished data). In contrast, an ectopic domain of FGF4 persists in the anterior apical ectodermal ridge (AER) (Figure 4H–4J). Thus the defects in limb patterning result from disruption of a step downstream of *Shh, Ptc1, gremlin,* and *Bmp4,* and upstream of *Fgf4*. This does not allow a specific molecular mechanism to be invoked, yet the combination of similar defects in both the limb and in the pattern of interneuron migration is strong evidence that the same molecular mechanism is disrupted in all three mutants.

### Migrating Cells Persist Abnormally in the IZ of 239, 251, and 275

**Figure 5:**
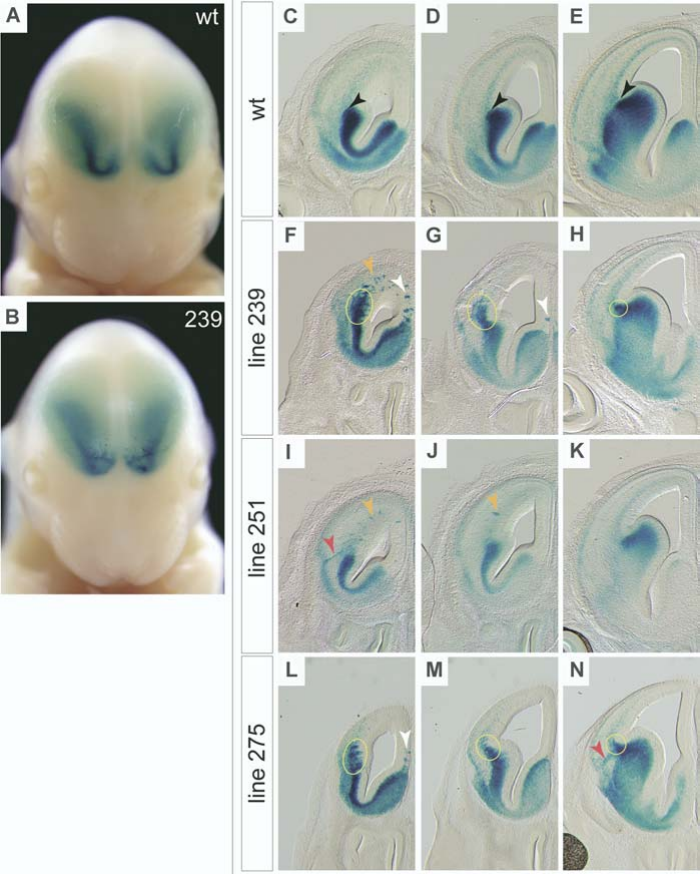
Line 239, 251, and 275 Mutants Have Similar Migration Defects (A) Frontal view of an E14.5 WT embryo. (B) Frontal view of an E14.5 embryo homozygous for the line 239 mutation. The stream of cells leaving the SVZ of the LGE is streaky and aggregated in the rostral cortex of the mutant. (C–E) A rostral-to-caudal series of coronal sections from an E14.5 WT embryo. WT embryos have diffuse cortical staining and a sharp boundary (black arrowheads) between the subcortical and the cortical telencephalon. (F–H) Coronal sections from E14.5 line 239 mutant forebrains. Yellow circles indicate the area of the cortical-subcortical boundary where a large excess of migrating cells can be seen in the IZ/SVZ area. Orange arrowhead indicates aggregated cells in the IZ/SVZ of the lateral wall of the cortex. White arrowheads indicate aggregates in the medial wall. The staining in the MZ appears normal. Defects become less apparent in the more caudal sections. (I–K) Sections from an E14.5 line 251 mutant embryo. Orange arrowheads indicate aggregates in the lateral wall of the cortex. In (I), a linear aggregate of stained cells can be seen extending from the IZ/SVZ to the MZ (red arrowhead). The cortical-subcortical boundary is well defined in line 251 mutants. (L–N) Sections from an E14.5 line 275 mutant embryo. Yellow circles indicate aberrant staining in the cortical-subcortical boundary region. The white arrowhead indicates aggregates in the medial wall of the cortex, and the red arrowhead points to a radially directed aggregate of cells extending from the IZ/SVZ to the MZ.

The 239, 251, and 275 mutants have clusters of LacZ-expressing cells in the IZ and/or SVZ of the rostral cortex, whereas more caudally, defects appear milder (Figure 5). In WT animals there is a clear demarcation, at the corticostriatal junction, between the SVZ of the dorsal LGE and the stream of cells migrating into the cortex (black arrowheads in Figure 5C–5E). The crispness of this boundary is lost in the 239 and 275 lines (yellow ovals in Figure 5F–5H and 5L–5N), although not in 251. In all three mutants, occasional linear aggregates of LacZ-expressing cells extend from the IZ/SVZ to the MZ (red arrowheads in Figure 5I and 5N).

**Figure 6:**
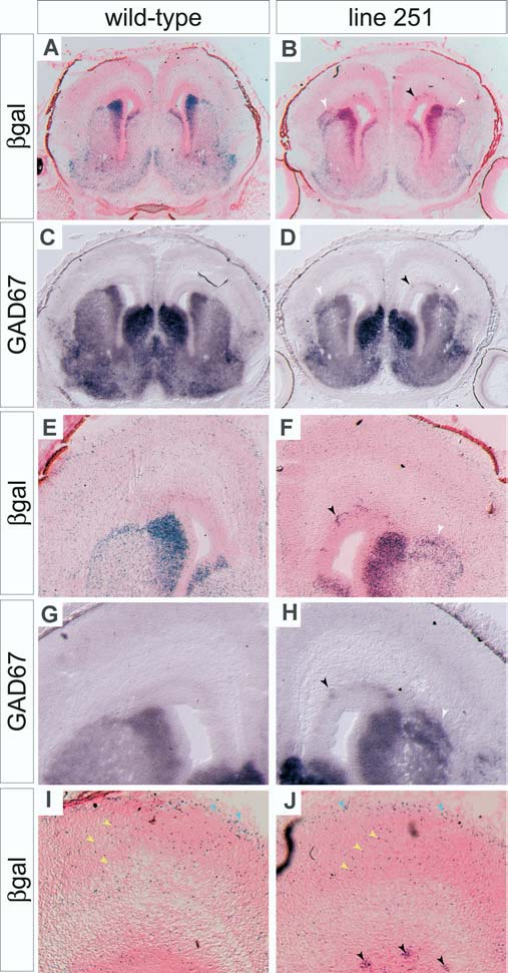
Dlx-LacZ and GAD67 Expression Show that Interneuron Precursors Persist in the IZ/SVZ of 251 Mutant Cortices (A) Coronal section through the forebrain of an E18.5 WT embryo stained for β-galactosidase and counterstained with nuclear fast red. (B) A similar section from a line 251 mutant embryo. Unusual accumulations of stained cells can be seen in the cortex just dorsal to the striatum (white arrowheads). Aggregates of cells in the IZ/SVZ can also be seen (black arrowhead). (C and D) Sections adjacent to those in (A) and (B) hybridized with a probe for *Gad67* mRNA. Arrowheads in (D) point to the same features that are seen in (B). (E–H) Higher-magnification views of the dorsal portions of the sections in (A–D). (I and J) Higher-magnification view of cortex. In the WT cortex (I), cells can be seen dispersed through the cortical plate (yellow arrowheads) and scattered through the MZ (blue arrowheads). Similar distributions of labeled cells can be seen in the mutant cortex (J). In contrast to the WT, however, aggregates of cells are found in IZ/SVZ (black arrowheads).

To study the effects of the mutations at later stages of cortical interneuron development, we examined near-term (E18.5) animals (Figure 6). We focused our analysis on line 251 and evaluated the expression of the *Dlx-LacZ* transgene and *Gad67*. As at E14.5, aggregation of *Gad67^+^; LacZ^+^* cells in the IZ/SVZ is a prominent feature of the phenotype (black arrowheads, Figure 6B, 6F, and 6J). Despite the severity of the defects observed in the IZ/SVZ, Dlx-LacZ–expressing interneuron precursors in the MZ and in the cortical plate do not form aggregates (yellow and blue arrowheads in Figure 6I and 6J), suggesting that the mutation inhibits the ability of migrating interneurons to leave the IZ/SVZ, but does not significantly impact other aspects of their migration.

### A Mutant with Defects in Invasion of the Cortex by Thalamocortical Axons

**Figure 7:**
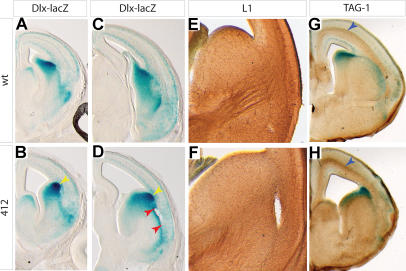
Corticostriatal Delamination and Lack of the Thalamocortical Projection in Line 412 Mutants (A–D) Coronal hemisections of E15.5 WT (A and C) and line 412 (B and D) mutant embryos stained for the *Dlx-LacZ* transgene. The cortex is thinner in 412 mutants, as can be seen in both the rostral (B) and the caudal (D) sections. Delamination of the corticostriatal boundary can be seen in the region between the red arrowheads in (D). (E and F) Immunostaining for L1 antigen labels the thalamocortical fibers in coronal hemisections from E14.5 WT (E) and mutant (F) embryos. The striatum and corticostriatal areas are shown. In the WT section, the thalamocortical fibers can be seen traversing the striatum through the internal capsule and coursing into the cortex. In the mutant section, a few fibers enter the striatum but do not traverse it to reach the cortex. Corticostriatal delamination can be seen as a hole in the right side of the section. (G and H) Immunostaining for TAG1 antigen (blue arrowheads) reveals corticofugal fibers.

In addition to the seven mutants described in the previous sections, six other mutants were isolated. Most of these have not been characterized in detail, but one, line 412, illustrates the range of phenotypes that the *Dlx-LacZ* transgene allowed us to identify. In line 412, the mutant phenotype was detectable in whole mount as a subtle but consistent defect near the cortical-subcortical boundary. Upon sectioning, this defect was revealed to be a delamination in the region of the external capsule (Figure 7A–7D). Thalamocortical fibers ordinarily enter the cortex at this point, and this defect can occur when fewer axon tracts cross this zone (Hevner et al. 2002). Indeed, the 412 mutant has fewer thalamocortical fibers traversing the corticostriatal boundary at E14.5, as evidenced by the reduced density of L1^+^ axons in this location (Figure 7E and 7F). Examination of late-stage embryos shows that the lack of cortical invasion by thalamocortical fibers at E14.5 is due to a persistent inhibition and not merely a delay in innervation (unpublished data). The IZ of the cortex often appears thinner in mutants, consistent with the absence of thalamocortical axons. Interestingly, TAG1^+^ corticofugal axons are still present in the cortex (Figure 7G and 7H). Analysis of markers for the dorsal thalamus, where the thalamocortical axons originate, and for the striatum, which they must traverse, does not reveal any obvious defects (A. Ashique, personal communication). This, together with the observation that corticofugal fibers appear to be intact, suggests that the mutation may disrupt a molecule that is directly involved in pathfinding by thalamocortical axons.

## Discussion

### Novel ENU-Induced Alleles

ENU overwhelmingly induces single-basepair substitution mutations. The mutant alleles that are produced often have relatively selective effects on protein function, and so provide valuable probes of the function of different protein domains. This is illustrated by two of the alleles described here, *Rfx4* and *Scrb1*. Each of these alleles is a missense mutation that appears to disrupt specific domains in a selective fashion.

The SCRB1 protein has two sets of recognizable domains: three amino-terminal LRRs and four, more carboxy-terminal, PDZ domains. The previously described allele of *Scrb1,* the *Circletail* allele, has a stop codon that terminates translation after the first two PDZ domains. In contrast, the line 90 allele of *Scrb1* is predicted to encode a charged lysine in place of a hydrophobic isoleucine on the outside of the third LRR domain (see Figure 2D and 2E). The homozygous phenotypes produced by the line 90 and the *Circletail* alleles are apparently identical, and both show strong genetic interactions with a mutation in *Str-1,* a cell-surface protein that regulates planar cell polarity in both flies and mammals. From this we can conclude that both the LRR and the PDZ domains are required for SCRB1's role in the establishment of epithelial polarity.

The missense allele of *Rfx4* is predicted to have very subtle effects on the structure of the protein. The mutation causes a proline to be substituted for a leucine residue in a dimerization domain of the protein. Proline residues are not compatible with α-helices, but in this case the substitution appears to be at the beginning of a β-turn that links two helices. Presumably, the substitution causes the linkage between the α-helices to be stiffened or abnormally constrained in some way. Whatever the exact molecular consequences are, the nature of the allele and the genetics suggest that the mutation prevents dimerization. Ordinarily RFX4 can dimerize with itself and with the related transcription factors RFX2 and RFX3 (Morotomi-Yano et al. 2002). If inactive dimers were produced by the missense allele, it would seem likely to cause a dominant phenotype, which it does not. Studies are underway that will test these ideas as well as determine whether the failure of the dorsal midline to form is the result of a loss of a dorsal signaling center or an inability of the cortical neuroepithelium to respond to those signals.

The *megalin* allele provides a contrast to these first two cases. Despite its molecular severity as a premature stop codon, its phenotypic consequences are less pronounced than the knockout allele. The knockout mutation produces early-head-fold–stage embryos with reduced neuroepithelium in the anterior midline (Willnow et al. 1996). These defects originate during gastrulation, and by mid-gestation result in holoprosencephaly. The absence of these early defects in the ENU-induced allele could be interpreted to mean that the defective protein retains some function and supplies the MEGALIN activity that is required during gastrulation. Alternatively, it is possible that the deleted region in the knockout is required for the proper expression of a neighboring gene and that the gastrulation phenotype does not reflect an early role for *megalin*.

In each of these three cases, the ENU-induced allele provides novel information about the role of the gene in cortical development and suggests avenues for further exploration. As we determine the molecular nature of the other mutations, it is likely that the selective nature of the ENU-induced alleles will provide important insights into the function of other proteins that regulate cortical development.

### Nature of the Tangential Migration Defects

The migration of immature interneurons has been followed using lipophilic dyes, tissue chimeras, transfection with a green fluorescent protein (GFP) expression vector, and, recently, using a *Gad67-GFP* knockin mouse (de Carlos et al. 1996; Anderson et al. 1997, 2001; Tamamaki et al. 1997; Denaxa et al. 2001; Polleux et al. 2002; Ang et al. 2003). These studies have identified tangentially migrating cells in both the IZ/SVZ and the MZ. Some studies have concluded that the cells in the IZ/SVZ and the MZ are two independent migratory streams (Lavdas et al. 1999). In contrast, the use of a *Gad67-GFP* knockin allele to follow interneuron precursors (Tanaka et al. 2003) led to the conclusion that the general pattern of cell migration is tangentially through the IZ and thence radially outward from the IZ to the MZ. Cells in the MZ also migrate tangentially but at a slower rate than those in the IZ (Polleux et al. 2002; Tanaka et al. 2003), in a process that may be a search for the proper location at which to invade the cortical plate (Ang et al. 2003). These two models for tangential migration produce different interpretations of the pattern of defects that we see in our novel mutants. In the first model, obvious defects in the IZ but not the MZ would imply that the mutations are disrupting a process used to regulate migration through the IZ but not through the independently regulated MZ. In the second model, the predominance of defects in the IZ/SVZ would indicate an inhibition at an early step of the migratory process. The presence of cell aggregates extending from the IZ to the MZ is consistent with this being the step that is defective, whereas the presence of properly dispersed cells in the MZ and cortical plate would indicate that the mutations inhibit, but do not completely block, this early step.

### Concordance of Limb Patterning and Migration Defects

Three out of the four tangential migration defects also result in anterior polydactyly. The association of limb patterning and neuronal migration defects has not been previously reported and, given that a great deal more is known about limb patterning than about tangential migration, it is tempting to speculate on what this may imply about the migration mutants. One possibility is that shared mechanisms are used to pattern the telencephalon and the limb. It is equally possible that genes that regulate patterning in the limb regulate cell migration decisions more directly. Mutations in an *arista-less* homolog, *Arx,* cause profound defects in interneuron migrations, and mutations in another *arista-less* homolog, *Alx4,* cause anterior polydactyly. However, as the limbs of *Alx4* mutants, unlike the 239, 251, and 275 mutants, have ectopic *Shh* expression in the anterior limb bud, it is unlikely that the mutations described here disrupt an *arista-less* pathway. *Shh* maintains the expression of *Fgf4* in the posterior AER of WT limb buds in a process that requires *gremlin*. Genes that act in this process downstream of *Bmp4* are plausible candidates for the line 239, 251, and 275 mutations.

### General Implications for Genetic Screens in the Mouse

The work described here demonstrates clearly that genetic screening strategies in the mouse need not be limited to general or broad-based phenotyping approaches; a focused genetic screening strategy can provide a powerful means of dissecting a specific aspect of mammalian biology. The strategy of broad-based approaches has been an effective one for the past several years as the collection of mutants using chemically induced mutations resurged in popularity. In other genetic systems, strategies have evolved from general toward focused screens that allow mutations affecting a specific process to be identified, and it is likely that mouse genetics will progress in a similar fashion. An additional concern with mice, however, is the cost of breeding and housing, which is higher than that for other model genetic organisms. We have shown, nonetheless, that a laboratory-based, focused screening strategy is a productive pursuit. The costs of such a screen could be shared by the careful combination of reporters so that screening for several distinct processes could be carried out at once. This general idea is highlighted by our isolation of mutations in which the growth and patterning of the cortex is defective. With the exception of line 90, the identification of these mutants benefited from the easy visualization of cortical size and structure by expression of the transgene. In several cases, the interneuron migration mutants being a good example, the identification of the mutant phenotype without the transgene would have been very unlikely. Independent reporters could be used to pursue the simultaneous identification of several different classes of mutants more directly. For example, reporters that label migrating neurons with GFP, and thalamocortical axons with β-galactosidase or alkaline phosphatase, would allow both migration and axonal pathfinding mutations to be identified in the same screen. Given the large number of reporter and indicator strains that have been made over the last few years and the powerful tools that exist for gene identification, it is clear that focused screens in the mouse will provide the resources to address many questions in the coming years.

## Materials and Methods

### 

#### Animals and breeding.

Male C57BL/6J mice were obtained from Jackson Laboratories (Bar Harbor, Maine, United States) and treated with three intraperitoneal injections of 100 mg/kg ENU (Sigma, St. Louis, Missouri, United States) spaced at 7-d intervals. Eight weeks after the last injection, the ENU-treated males were set up in breeding pairs with FVB/NJ females homozygous for the *Dlx-LacZ* transgene. Male offspring of this cross (G1 males) were backcrossed to the transgenic line, and female offspring (G2 females) were saved. For each line, one to six of the G2 females were backcrossed to their fathers to generate timed pregnancies. Embryos were harvested either 13 d (the first 100 lines) or 14 d (all subsequent lines) after the vaginal plug was identified.

#### Screening.

Embryos were dissected in phosphate-buffered saline (PBS) and fixed for 45 min at room temperature in 4% paraformaldehyde (PFA) in PBS. Subsequently, embryos were washed in detergent rinse (0.1 M phosphate buffer [pH 7.3], 2 mM MgCl_2_, 0.01% sodium deoxycholate and 0.02% Nonidet P-40). Embryos were then stained for 48–72 h at room temperature on a rocking platform using X-gal as a substrate for the detection of β-galactosidase activity. Staining was terminated after visual inspection by repeated washing in PBS. Embryos were then fixed again and stored in 4% PFA in PBS until further examined. All litters were examined in whole mount and approximately one-half were selected for sectioning on a vibratome (VT1000S; Leica, Wetzlar, Germany). For sectioning, the heads of all pups from a litter were separated from the torsos and mounted aligned in the same orientation in an agarose block. Sections 100 μm thick were collected in PBS and mounted in Kaiser's glycerol gelatin (Merck, Darmstadt, Germany) on slides. All phenotypes reported here were seen in more than ten litters resulting from both G1 male × G2 female and G2 male × G2 female crosses.

#### Histology.

Whole-mount in situ hybridization was carried out according to Henrique et al. (1995). *Gad67* in situ hybridization on sections was carried out following standard protocols (Dagerlind et al. 1992) with a digoxigenin-labeled antisense RNA probe generated by in vitro transcription using a plasmid obtained from Brian G. Condie (Maddox and Condie 2001). In brief, embryos were dissected in diethylpyrocarbonate-treated PBS, and the heads were removed and fresh-frozen on dry ice. Tissue was sectioned at 20 μm on a cryostat (Leica CM3050). Slide-mounted cryosections were warmed to room temperature and fixed in 4% PFA in PBS. Deacetylation was performed for 10 min by immersion in 0.1 M triethanolamine containing 25 mM acetic anhydride followed by rinsing in 2× saline sodium citrate and dehydration through an increasing alcohol series (60%, 75%, 95%, and 100%). Sections were hybridized with the riboprobe under stringent conditions (50% formamide, 10% dextran sulfate, 20 mM Tris-HCl, 0.3 M NaCl, 5 mM EDTA, 0.02% Ficoll 400, 0.02% polyvinylpyrrolidone, 0.02% BSA, 0.5 mg/ml tRNA, 0.2 mg/ml carrier DNA, and 200 mM DTT) for 16–20 h at 63 °C. After hybridization, sections were washed four times in 4× saline sodium citrate solution and incubated for 30 min in RNase buffer (10 mM Tris-HCl [pH 7.5], 0.5 M NaCl, and 5 mM EDTA [pH 8.0]) containing 20 μg/ml RNase A at 37 °C. High-stringency washes were performed twice for 30 min at 63 °C. Incubation with the anti-digoxigenin antibody (Roche, Basel, Switzerland) was carried out in maleic acid buffer (100 mM maleic acid, 150 mM NaCl, and 1% blocking reagent [Roche]). Finally, staining was performed with BM purple substrate (Roche) terminated by repeated washes in PBS, and sections were coverslipped using Kaiser's glycerol gelatin. Nuclear fast red counterstaining for X-gal–stained sections was performed with pre-made staining solution (Vector Laboratories, Burlingame, California, United States). Staining was differentiated in 70% ethanol.

Immunohistochemistry using anti-TAG1 (Developmental Studies Hybridoma Bank, University of Iowa, Iowa City, Iowa, United States) and anti-L1 (Chemikon, Mönchengladback, Germany) antibodies was performed on 100-μm free-floating sections following the instructions and using the reagents of the Vectastain ABC Kit (Vector). Primary antibodies were used at a dilution of 1:500. Horseradish peroxidase activity from secondary antibodies was revealed by diaminobenzidine as a substrate in staining buffer (0.5 mg/ml diaminobenzidine, 20 mM sodium cacodylate, and 30 mN acetic acid).

#### Mapping.

Initial linkage was established using 12 DNA samples from both carriers and mutant embryos. We scored a set of 82 simple sequence repeat markers. The panel of markers was selected from the set at the Center for Inherited Disease Research Website (http://www.cidr.jhmi.edu/mouse/mouse.html). Markers were chosen that could be scored easily on agarose gels. All chromosomal assignments reported are the result of LOD scores significantly greater than three. The intervals that are listed in Table 1 were derived following the initial establishment of linkage by haplotype analysis that included additional recombinant chromosomes from mutant animals or obligate carriers. The phenotypes of 239 and 275 are very similar, and the fact that they map to the same interval likely reflects allelism. The localization of both 351 and 357 to the same interval seems more likely to be coincidental, because the phenotypes are quite different.

#### Sequencing.

For sequencing of *Rfx4* and *Scrb1* genes, RT-PCR samples were prepared from cDNA prepared using E10.5 to E16.5 embryos. For *megalin,* exons were amplified using genomic DNA samples. Primer sequences are available upon request. In both cases sequencing was done by the University of California, Berkeley, DNA Sequencing Facility.

## Supporting Information

Figure S1 Cortical Size Is Altered in Lines 152 and 351 (A) Dorsal views of the cortex of WT (left) and line 152 mutant (right) embryos stained for expression of the Dlx-LacZ transgene. The line 152 mutation reduces the size of the cortex.
(B and C) Coronal sections through the cortex of E14.5 WT (B) and line 351 mutant (C) embryos. The cortex has a characteristic high-domed shape and is thinner in the mutant.
(D and E) Dorsal (D) and ventral (E) views of adult brains of WT (left) and line 351 mutant (right) brains. The cortex is overall larger in the mutant than in the WT. The olfactory bulbs are present in the mutant but are tucked under the cortex, making them less visible.
(9.1 MB TIF).

Figure S2 Central Nervous System Defects and Cleft Upper Jaw in Lines 366 and 357 (A) Lateral view of an E14.5 WT embryo.
(B) The cleft upper jaw of a line 366 mutant is visible at the left. The telencephalon, including the cortex, is significantly shortened along the rostrocaudal axis.
(C and D) Lateral and front views of the cleft and reduced upper jaw of an E18.5 embryo homozygous for the line 366 mutation.
(E and F) Lateral views of WT (E) and line 357 homozygote (F) embryos at E13.5 showing the reduced telencephalon and relatively expanded midbrain.
(G) A front view of the embryo in (F) shows the cleft upper jaw.
(H and I) Sagittal sections through E13.5 WT (H) and line 357 (I) embryos. The overgrown midbrain in the mutant embryo has forced the neuroepithelium into folds.
(9.9 MB TIF).

Figure S3 Line 407 Mutants Have Dorsoventral Defects in the Cortical Primordia and Facial Midline Defects (A) Lateral view of an E14.5 mutant embryo showing edema and hemorrhage suggestive of vascular defects. Frontal views of WT (B) and mutant (C) embryos illustrate the narrowed frontonasal process, maxilla, and mandible of the mutant. (D) shows coronal hemisections in a WT embryo. (E) shows the accumulation of Dlx-LacZ–positive cells in a SVZ-like area dorsal to the LGE.
(4.3 MB TIF).

### Accession Numbers

Accession numbers of the genes discussed in this paper are available at LocusLink (http://www.ncbi.nih.gov/LocusLink, and are as follows: *Alx4* (11695), *Arx* (11878), *Bmp4* (12159), *Dlx5/6* (13395/13396), *Fgf4* (14175), *Gad67* (14415), *gremlin* (23892), *Hoxd13* (15433), *L1* (16728), *Lrp2* (14725), *Ltap/Lpp1* (93840), *Ptc1* (19206), *Rfx4* (71137), *Scrb1* (105782), *scribbled* (44448), *Shh* (20423), and *Tag1* (21367).

## Abbreviations

AER: apical ectodermal ridge
A-P: anterior-posterior
E[number]: embryonic day [number]
ENU: ethyl-nitroso-urea
GFP: green fluorescent protein
IZ: intermediate zone
LGE: lateral ganglionic eminence
LRR: leucine-rich repeat
MZ: marginal zone
P[number]: postnatal day [number]
PBS: phosphate-buffered saline
PFA: paraformaldehyde
*Shh*: *sonic hedgehog;* SVZ
WT: wild-type
