# Supplementary figures and images for: A Focused and Efficient Genetic Screening Strategy in the Mouse: Identification of Mutations That Disrupt Cortical Development
Source: PLoS Biol. 2004 Aug 17;2(8):e219. doi: 10.1371/journal.pbio.0020219 (PMC509294; doi:10.1371/journal.pbio.0020219)

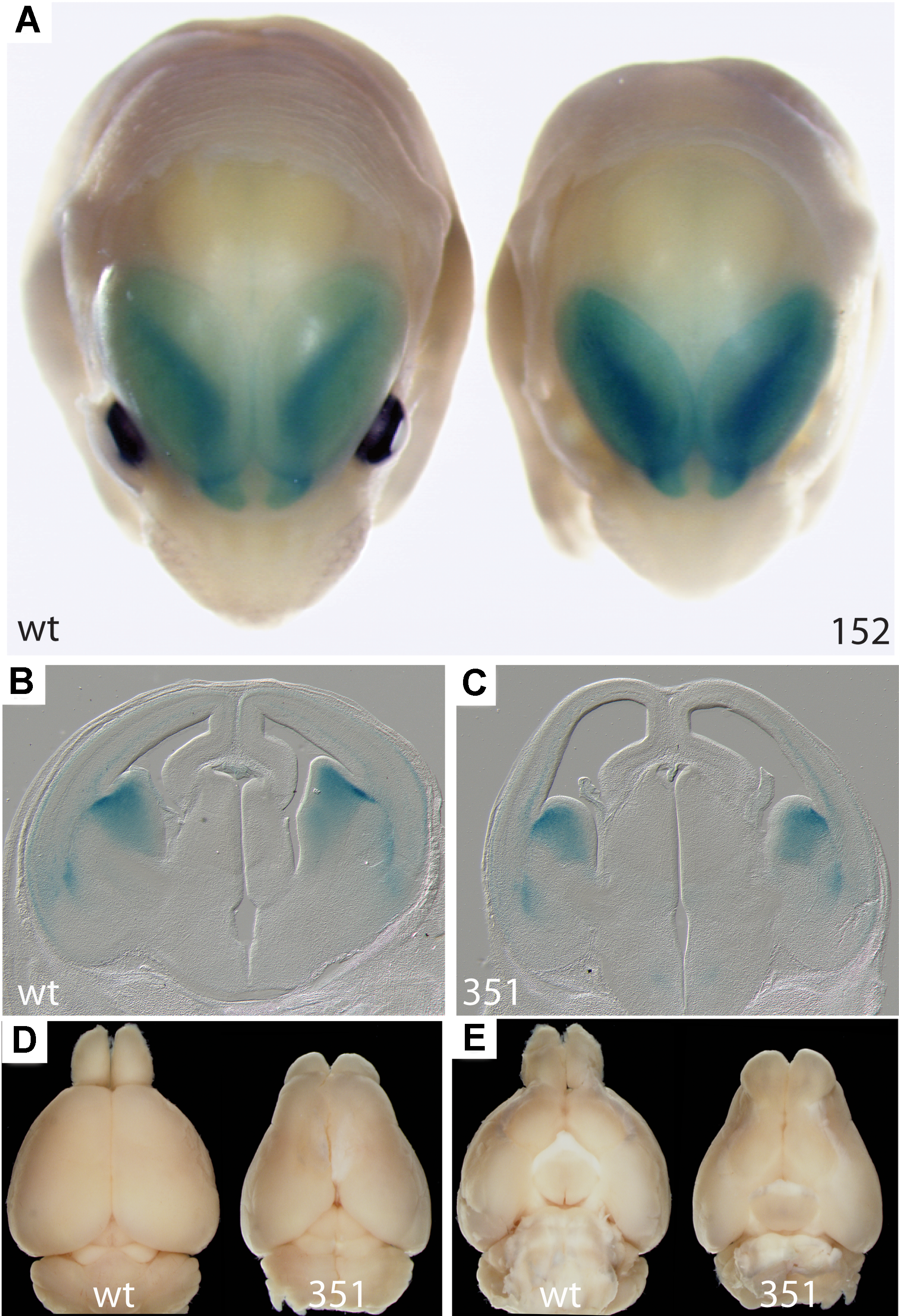

Supplement: Figure S1 — (A) Dorsal views of the cortex of WT (left) and line 152 mutant (right) embryos stained for expression of the Dlx-LacZ transgene. The line 152 mutation reduces the size of the cortex. (B and C) Coronal sections through the cortex of E14.5 WT (B) and line 351 mutant (C) embryos. The cortex has a characteristic high-domed shape and is thinner in the mutant. (D and E) Dorsal (D) and ventral (E) views of adult brains of WT (left) and line 351 mutant (right) brains. The cortex is overall larger in the mutant than in the WT. The olfactory bulbs are present in the mutant but are tucked under the cortex, making them less visible. (9.1 MB TIF). [file pbio.0020219.sg001.tif]

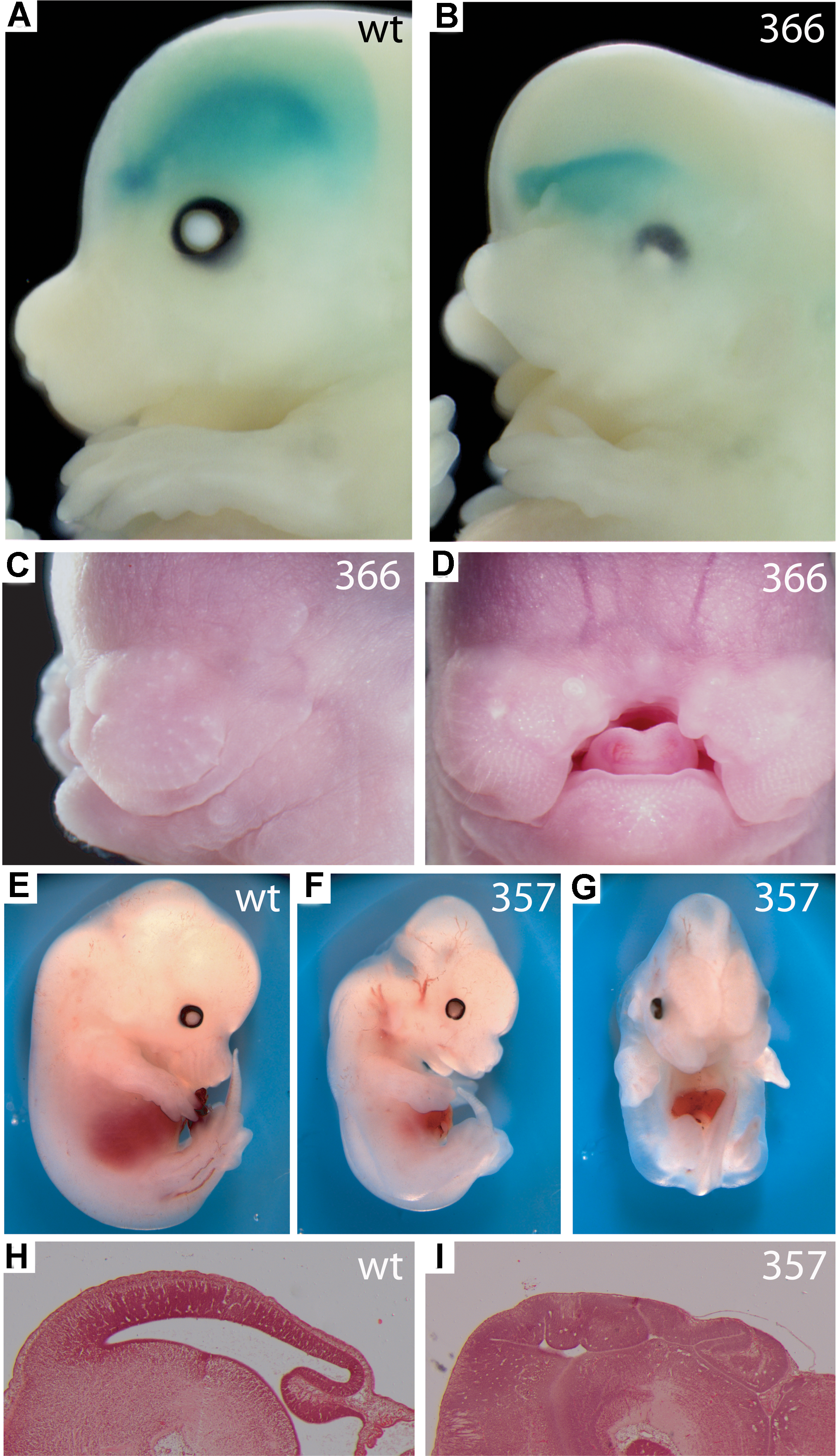

Supplement: Figure S2 — (A) Lateral view of an E14.5 WT embryo. (B) The cleft upper jaw of a line 366 mutant is visible at the left. The telencephalon, including the cortex, is significantly shortened along the rostrocaudal axis. (C and D) Lateral and front views of the cleft and reduced upper jaw of an E18.5 embryo homozygous for the line 366 mutation. (E and F) Lateral views of WT (E) and line 357 homozygote (F) embryos at E13.5 showing the reduced telencephalon and relatively expanded midbrain. (G) A front view of the embryo in (F) shows the cleft upper jaw. (H and I) Sagittal sections through E13.5 WT (H) and line 357 (I) embryos. The overgrown midbrain in the mutant embryo has forced the neuroepithelium into folds. (9.9 MB TIF). [file pbio.0020219.sg002.tif]

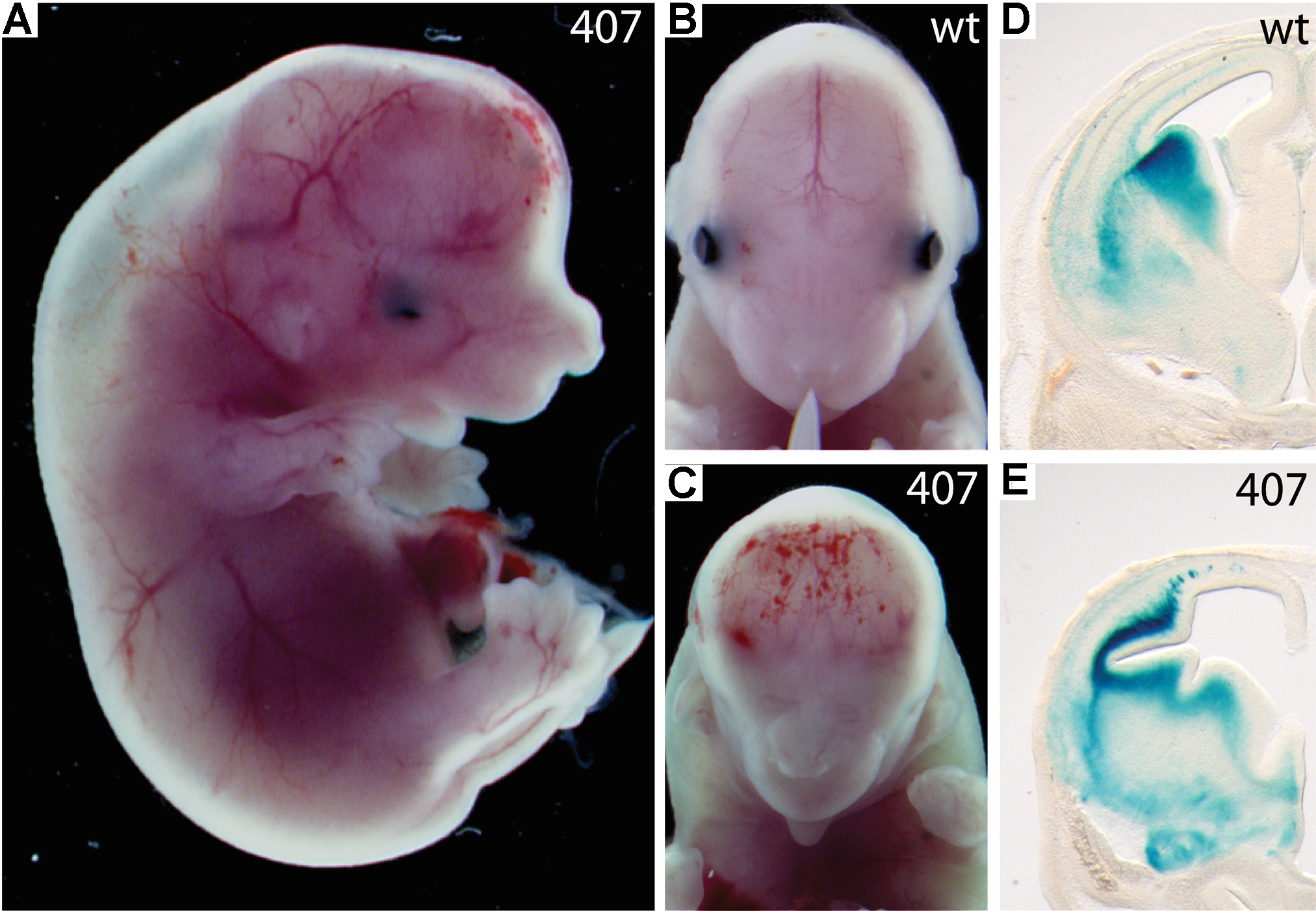

Supplement: Figure S3 — (A) Lateral view of an E14.5 mutant embryo showing edema and hemorrhage suggestive of vascular defects. Frontal views of WT (B) and mutant (C) embryos illustrate the narrowed frontonasal process, maxilla, and mandible of the mutant. (D) shows coronal hemisections in a WT embryo. (E) shows the accumulation of Dlx-LacZ–positive cells in a SVZ-like area dorsal to the LGE. (4.3 MB TIF). [file pbio.0020219.sg003.tif]
